# Theory in quality improvement and patient safety education: A scoping review

**DOI:** 10.1007/s40037-021-00686-5

**Published:** 2021-10-05

**Authors:** Joanne Goldman, Andrea Smeraglio, Lisha Lo, Ayelet Kuper, Brian M. Wong

**Affiliations:** 1 Centre for Quality Improvement and Patient Safety, Temerty Faculty of Medicine, University of Toronto, Toronto, Ontario Canada; 2 The Wilson Centre, Toronto, Ontario Canada; 3 Department of Medicine, University of Toronto, Toronto, Ontario Canada; 4 Department of Medicine, Oregon Health & Science University, Portland, OR USA; 5 Division of Hospital & Specialty Medicine, Portland Veterans Administration Medical Center, Portland, OR USA; 6 Sunnybrook Health Sciences Centre, Toronto, Ontario Canada

**Keywords:** Quality improvement, Patient safety, Education, Theory, Scoping review

## Abstract

**Introduction:**

Theory plays an important role in education programming and research. However, its use in quality improvement and patient safety education has yet to be fully characterized. The authors undertook a scoping review to examine the use of theory in quality improvement and patient safety education.

**Methods:**

Eligible articles used theory to inform the design or study of a quality improvement or patient safety curriculum. The authors followed scoping review methodology and searched articles referenced in 20 systematic reviews of quality improvement and patient safety education, or articles citing one of these reviews, and hand searched eligible article references. Data analysis involved descriptive and interpretive summaries of theories used and the perspectives the theories offered.

**Results:**

Eligibility criteria were met by 28 articles, and 102 articles made superficial mention of theory. Eligible articles varied in professional group, learning stage and journal type. Theories fell into two broad categories: learning theories (*n* = 20) and social science theories (*n* = 11). Theory was used in the design (*n* = 12) or study (*n* = 17) of quality improvement and patient safety education. The range of theories shows the opportunity afforded by using more than one type of theory.

**Discussion:**

Theory can guide decisions regarding quality improvement and patient safety education practices or play a role in selecting a methodology or lens through which to study educational processes and outcomes. Educators and researchers should make deliberate choices around the use of theory that relates to aspects of an educational program that they seek to illuminate.

**Supplementary Information:**

The online version of this article (10.1007/s40037-021-00686-5) contains supplementary material, which is available to authorized users.

## Introduction

Quality improvement and patient safety (QIPS) education plays a critical role in developing healthcare professionals with the knowledge, skills and attitudes to improve healthcare systems. Over the past 15 years, systematic reviews of QIPS education programs provided useful syntheses of QIPS education [1–5]. These reviews summarized target audiences, curricular content, teaching methods, and the positive impact of QIPS education on learners’ knowledge and skills, as well as some evidence of impact on learners’ behaviours and process improvement. They also listed learner, faculty, training program and health system factors that require attention when implementing QIPS education programs. While these reviews have shaped education practices, their focus has generally not foregrounded the use of theory. Yet theory is widely accepted as playing an important role in the design and study of education programs, as well as research on education more generally [6–8]. Thus, understanding the ways that theory has informed, and can inform, QIPS education is essential to advancing the field.

Theory can be defined as *“an abstract description of the relationships between concepts that help us to understand the world”* [9, p. 990]. Educators use theory to plan, and improve, teaching approaches and curricular design [10], enabling them to *“move beyond ‘cookbook’ methods based on experience …”* [11, p. 184]. Mann [10] effectively compared the use of theory in education to the prescription of a drug. A clinician can prescribe a drug based only on the information that it will work. However, insight about its mechanism of action is needed to understand how the drug works, its interaction with other drugs and why it might not work, or to create better drugs. Similarly, knowing how an education intervention works and how learners might interact with it in a particular context allows for an optimal approach to be chosen. Furthermore, understanding why an education intervention is not effective can contribute to its adaptation and iterative improvement. Theory thus allows for a more reflective rationale and deliberate approach to planning and implementing education programs [12–14].

Education researchers’ use of theory can be conceptualized in various ways, each providing a different lens to appreciate its potential value [6, 15]. Brown et al. [15] propose one taxonomy, describing six uses for theory in a research study. These are: (1) to align theory with a perspective taken in the paper, (2) to identify the research problem, (3) to serve as a vehicle for an idea, (4) to provide a methodological tool, (5) to provide an approach for interpretation and (6) to be a study’s object of examination. As Brown et al. note, the choice of theory for a particular role and how to use it depends on the field and the audience.

Clearly theory can play different roles; the above descriptions allow us to be more explicit about how we recognize it (as readers) and make use of it (as educators and researchers) in different contexts. QIPS education publications increasingly refer to theory, but the extent to which theories are being used in this field and the nature of those theories has not yet been well-defined. We therefore undertook a scoping review to examine the extent, range and nature of the use of theory in QIPS education [16]. The purpose of this review is to answer the following questions: (1) To what extent are theories being used in QIPS education? (2) What theories have been used in QIPS education? (3) How have theories been used in QIPS education? (4) What types of perspectives do identified theories offer about QIPS education? and (5) What theoretical gaps exist in QIPS education?

## Methods

We used a scoping review methodology as defined by Colquhoun [17], given the exploratory nature of our research questions with the goals of mapping out the use of theory in QIPS education and identifying gaps in the literature. We followed the framework of Arksey and O’Malley [18] as enhanced by Levac et al. [19] and further refined by the Joanna Briggs Institute [20]. The PRISMA Extension for Scoping Reviews was used in the development of this manuscript [16] (see Part Three of the Appendix in the Electronic Supplementary Material (ESM)).

### Eligibility criteria

We included English-language peer-reviewed articles that (a) described a QIPS curriculum or explicit education about QIPS and (b) used theory to inform how it was developed or studied. We included education programs that involved students or practitioners from any healthcare professional group and occurred in any education or healthcare organizational setting. An article met the ‘QIPS curriculum’ criteria if it was a description and/or study (using quantitative, qualitative or mixed methods) of any kind of organized learning of QIPS. A curriculum was defined as QIPS if the authors used these terms or concepts associated with the field (e.g., health systems science, human factors) and topics included processes and tools associated with the field (e.g., Model for Improvement, medical errors, incident reporting). An article met the ‘use of theory’ criteria if it cited a theory used and provided some detail about the theory and the connection between the theory and the education curriculum development or study. We used the definition of theory as a *“set of interrelated constructs (concepts), definitions, and propositions that present a systematic view of phenomena by specifying relations among variables with the purpose of explaining and predicting phenomena”* [21, p. 9]. We excluded articles that mentioned theory superficially (e.g., in a few words with no further elaboration) or that used learning models or frameworks which we did not classify as theory (e.g., Kirkpatrick model). We also excluded articles if patient safety was just used as a rationale for the education program, QI methods were used to improve the education program, or the article reported on building skills related to QIPS (e.g., professionalism and interpersonal skills) but not about learning QIPS itself.

### Search strategy

A number of published systematic reviews have already identified QIPS education articles targeting learners across the learning curriculum from various health professions. Therefore, we used the keywords ‘curriculum’, ‘patient safety’, ‘quality improvement’ and ‘review’ in PubMed, ERIC and EMBASE (Database search strategy in ESM Appendix, Part One) to identify 20 systematic reviews of QIPS education (List of systematic reviews in ESM Appendix, Part Two). We performed a search in April 2018, which was updated in July 2020. We included all articles from these reviews as well as articles published after these reviews that cited one or more of these reviews, using Web of Science to capture these latter articles. We looked through reviews identified in the results to identify further relevant studies. Additionally, we hand searched the references of the included articles and journals which had published two or more of the included articles.

### Article review process

We used a two-stage review process to select included articles. The first focused on identifying articles about QIPS curricula. Three authors (JG, AS, BW) reviewed an initial set of 10 abstracts to ensure agreement regarding inclusion and exclusion criteria relating to QIPS curricula. Two authors (any combination of JG, BW, AS) independently reviewed the remaining abstracts. An 82–84% agreement between the reviewers occurred at this first stage. Articles identified as being about a QIPS curriculum by at least one reviewer, and articles where a determination could not be made, were promoted to the second review stage. The same three authors reviewed 10 promoted articles in full-text to ensure agreement regarding inclusion and exclusion criteria relating to use of theory. Two authors (JG plus either AS or BW) independently reviewed the remaining full-text articles to identify those that were about a QIPS curriculum and included use of theory. An 89–93% agreement between the reviewers occurred at this second stage. We resolved disagreements by seeking input from the third reviewer, and where necessary, from a fourth author (AK).

### Data extraction and synthesis

We developed and revised a data abstraction form after piloting on three randomly selected articles. We tested it on three more randomly selected articles and revised again. Two study authors (JG plus either AS or BW) independently abstracted the following data from included articles: authors, journal, publication year, country of origin, education setting, curriculum (curriculum objectives, teaching methods, duration, topics, participant details), research (study objectives, study design, Kirkpatrick evaluation level, other outcomes), and theory (theory used, purpose of use of theory, theory references). The two study authors reconciled data extraction details.

We did not examine the articles for methodological rigour as the purpose of this review was to map out the ways in which theory is being used in QIPS education rather than to make judgements as to the quality of such studies. Furthermore, assessing the rigour with which studies used theory is inherently problematic because the diverse disciplines represented in the QIPS education literature draw on different (and sometimes contradictory) definitions of research rigour and quality.

For data synthesis and interpretation, we began by summarizing descriptive details of each article. We then created narrative syntheses of how theory was used in each article and organized theories into various categories, including the types of theory, how theory was used (to develop or study QIPS education program) and the types of perspectives the theories offered. Articles identified as ‘superficial mention of theory’, and not included in our final group of papers, were classified by the type of theory listed.

## Results

We screened 1126 titles and abstracts (stage 1) of articles identified through the 20 review articles and identified 43 articles from 5 reviews not part of the original search. We promoted 547 articles for full-text review (stage 2) (see ESM, Fig. S1).

Twenty-six articles met eligibility criteria, with an additional four articles identified through hand search, for a total of 30 articles. Three of these articles [22–24] were based on data from one study and reported on the same theory. Therefore, we included only one of these [22], leading to a total of 28 articles (see ESM, Table S1). One hundred and two articles made superficial mention of use of theory (See ESM, Table S2), not meeting our inclusion criteria.

Articles were published between 2009 and 2020, apart from one published in 1995. Twelve articles focused on PS education, twelve on QI education and four on both QI and PS education. Most of the journals that published these articles were focused on health professions education (46%) and QIPS (36%). Most of the QIPS education programs took place in Europe (50%) and the US (36%), with a minority in Canada, Australia and South Africa. Most studies included undergraduate medicine, nursing, pharmacy and physiotherapy students (43%) or postgraduate medical trainees, plus medical staff in some cases, from a range of specialty training programs (39%). Most of the educational settings in the articles were university-based professional training programs (53%) or hospital/medical centres (32%).

### What theories are being used in QIPS education

The theories used fall into two broad, though overlapping, categories: learning theories (*n* = 20) and social science theories (*n* = 11). Table S3 (See ESM) summarizes definitions of theories reported and details of their use.

Learning theories are specifically concerned with how learning occurs, including what *“drives and impedes learning”* [25] and the strategies and environments that affect learning [10]. Learning theories used in the 15 articles were from the following categories: cognitive (*n* = 11), which included spaced education, team competition and testing effect, theory of intrinsic motivation, reflective learning, Kolb’s experiential learning, theory of attitude-relevant knowledge, self-regulated learning, and cognitive transfer [26–36]; sociocultural (*n* = 3), which included Eraut’s theory of formal and informal learning, Senninger’s theory of learning, and community of practice [22, 33, 37]; transformative (*n* = 7), which included Mezirow’s theory of transformative learning and Sandars’ critical reflection [35, 38–43]; and organizational (*n* = 1), which included psychological safety [44].

Social science theories refer to a wide range of disciplines (e.g., anthropology, economics, psychology) concerned with human behaviours and their social and cultural aspects. The ten articles that drew upon social science theories were from the fields of psychology (*n* = 7), which included theories of planned behaviour, behavioural psychology, self-determination, self-efficacy, as well as activity theory that has origins in psychology [31, 38, 45–49]; sociology (*n* = 2), which involved Bourdieu’s concepts of field, habitus and capital and the sociological theory of professions [50, 51]; and philosophy (*n* = 2), with both articles using realist evaluation [38, 52].

### How theories are used in QIPS education

Articles demonstrated the use of theories in two main ways, namely, the design (*n* = 12) or the study (*n* = 17) of QIPS education. While many articles could fit into both categories, such as an article that described the use of theory in the development of a curriculum and then evaluated the effectiveness of the educational approach, we classified each article by its dominant focus. One exception is De Feijter et al. [31], who utilized one theory to design their patient safety learning and a second one to study it.

In designing QIPS education, the twelve articles described the use of theory to inform decisions regarding education format [26–28, 32] (e.g., online learning) and teaching strategies [30, 31, 33, 36, 39] (e.g., reflection), as well as strategies that attend to facilitators and barriers of QI project-based learning [47], faculty engagement [50] and the learning environment [44]. For example, one program developed an online, quiz-based QIPS education program, based on the psychological phenomena of knowledge presentation, repetition, spacing and testing impacting on learners’ retention and retrieval of information [28]. Another study used transformative learning theory, along with a conceptual framework of empathy and moral development, in their development of a program that involved a patient group sharing stories about inadequate care and its causes, followed by facilitated discussion between the patients and the junior doctors to allow for the doctors to reflect on the narrative and explore their own attitudes and beliefs about patient safety [39].

In 17 articles, theory was mainly used to study QIPS education programs. Seven articles used theory to help predict or understand learners’ reactions to QIPS education and impact of the education on learner’s knowledge, attitudes and behaviours [31, 34, 35, 38, 45, 46, 48]. For example, Jansma et al. [45] used theory of planned behaviour to underpin their study of a two-day patient safety course for residents. Based on the theory that intention is the immediate antecedent of behavioural change, they requested participants to formulate an action to improve patient safety following the course, and then conducted follow-up interviews three months later to examine actions and understand gaps between intentions and behaviours. In six articles, theory provided an analytical approach for interpreting QIPS learning processes [22, 29, 37, 43, 49, 51]. For example, de Feijter et al. used activity theory as an analytic framework to highlight potential difficulties of learning about patient safety in the complex system of workplace learning [49]. In three studies, theory was the object of examination; these included examinations of the processes and impacts of reflection [41, 42] and how QI project work can lead to transformative learning [40]. Two studies used realist evaluation as their methodological approach to study questions of ‘what works, in which circumstances and for whom’ in QIPS education programs [38, 52].

### What types of perspectives do identified theories offer about QIPS education?

Articles that used theory to inform the design of QIPS education turned most frequently to learning theories, with cognitive theories being the single largest group of theories represented. However, articles reported on a range of learning theories stemming from different orientations (e.g., cognitive, transformative, organizational) [26–28, 30–33, 36, 39, 44], with two additional studies drawing upon psychological [47] and sociological theories [50] that demonstrate the potential for expanding beyond learning theories to inform QIPS education design. There were also examples of articles that focused on a particular teaching approach, but which drew upon different theories. For example, the use of reflection was connected to several different theories, such as Schon’s cognitively oriented reflection learning theory or Mezirow’s transformative learning theory. Taken together, these findings suggest that there is no single dominant theoretical lens through which to view QIPS educational design.

Articles that used theory as a lens to interpret QIPS education drew upon different types of theories including sociocultural learning theories, activity theory with origins in psychology, the cognitively oriented learning theory of intrinsic motivation and sociology of professions theory [22, 29, 37, 49, 51]. These articles examined different types of learning and social processes (e.g., formal and informal acquisition of knowledge, zones for learning, professional socialization) occurring across different types of educational activities and settings (e.g., workplace based learning, QI projects, undergraduate or continuing professional development curriculum), and highlighted the complexity of delivering QIPS education and the interplay of the various factors that influence learning processes and outcomes. Articles that used theory to help predict or understand the impact of QIPS education largely drew upon theories from psychology [30, 45, 46, 48], with less attention given to social and organizational factors that affect changes in behaviour. A few studies also provide examples of how to build evidence to support theoretically informed learning approaches in QIPS. For example, Wittich et al. [42] explored transformative learning processes by studying the associations between resident doctors’ reflections on QI opportunities and the quality of their QI project proposals. Finally, the two studies [38, 52] that used a realist evaluation illustrate how theory can influence methodological approaches in QIPS education research.

### What theoretical gaps exist in QIPS education?

Many studies (*n* = 102) made superficial mention of theory, which seems to indicate interest in using theory to guide education planning or study. However, the articles were limited by having only a cursory description of the theory, with a few words and no further elaboration (ESM, Table S2). Many of these articles (*n* = 57) referred to experiential and/or active learning, or experiential learning in combination with another theory (e.g., adult learning, reflective practice) (*n* = 21).

## Discussion

This scoping review describes the range of learning and social science theories that have been used to design and study QIPS education, providing insights into the role of theory in QIPS education and future directions. The findings show how theory can be relevant to QIPS educators and help guide teaching approaches and curriculum design [10, 11]. The understandings based on theory provide knowledge of the ‘mechanism of action’ that enable more deliberate QIPS education planning. An emerging concern in QIPS education is the limited attention provided to the concept and process of experiential learning [53]. Our findings reflect this concern, evidenced by the large number of articles we excluded that made superficial mention of experiential learning. We note, however, that experiential learning is an umbrella term that includes a range of theory types, some of which are found in this review, such as Kolb’s experiential learning theory, activity theory, and reflective practice [54]. The articles in this review provide insights into how educators might optimize experiential learning approaches by incorporating a more theoretically informed approach to the construction of *“knowledge and meaning from real-life experience … in a context relevant to learners’ own future careers”* [55, p. 161].

This scoping review is also helpful in showing how theory is integral to the study of QIPS education. A comparison of our findings to Brown et al.’s [15] six uses of theory shows that the articles in our review used theory particularly to provide a methodological tool, for interpretation and as an object of examination. However, the other three categories—aligning theory with a perspective taken in the paper, to identify the research problem and to serve as a vehicle for an idea—could also be relevant. Researchers of QIPS education can use theory more deliberately at different stages of a study by drawing upon this and other related frameworks. For example, building on de Feijter et al.’s research findings [49] about the contradicting priorities between patient safety and learning to be a physician, a researcher could identify the research problem of the general use of the term experiential learning in QIPS education and then use activity theory to interpret the effects of an education program designed to provide strategies to practice safely [54].

There is no single best approach to the use of theory; rather, a theory is chosen for a particular purpose. Educators and researchers in QIPS make deliberate choices around theories used based on their own theoretical orientation and interests, and the objectives of their education program or study. Educators also cannot expect a single theory, or even multiple theories, to provide a complete understanding of a particular phenomenon related to QIPS education. While helpful in illuminating a particular aspect of teaching and learning, selected theories inevitably leave other processes unknown [12, 56]. The range of theories reported in this review—which included cognitive, sociocultural and transformative learning theories, as well as theories from psychology, sociology and philosophy—is consistent with this broader understanding for how theories are being used in health professions education.

The range of theories to draw upon in the future is immense, and we therefore provide several examples to highlight potential opportunities. Critical theories that bring attention to issues of power and hierarchy could, for example, be used to study hidden curricula [57] in QIPS education and inform communication skills training related to speaking up in patient safety. Social-cognitive theory, which considers the interactions between personal factors, behavioural factors and environmental factors [58], could provide a lens for studying how these factors affect an individual’s learning experience in QIPS education. Theories of adaptive expertise posit that there are two ways that knowledge is used in practice: as a repertoire of solutions to be applied (efficiency) or as the starting point for the creation of new solutions (innovation); these can be used to design education that allows learners to respond to changing and complex healthcare contexts as they apply QI tools in practice [59]. These are three broad theoretical directions amongst many, and we can imagine many other theories that could be applied to QIPS education.

We did not assess the rigour with which theory was used for reasons stated earlier. That said, we offer some thoughts relating to quality issues in the use of theory in QIPS education. The first relates to the depth in which authors describe the use of theory. Brown et al.’s [15] description of the wide variation in the *“prominence of the role”* that is given to theory, from a *“cameo character”* through to a *“major character”*, reflects the articles included in our review. The former refers to an appearance of theory in a small number of sentences in the introduction or discussion and the latter to theory being a significant presence throughout the paper. We acknowledge that some articles categorized as superfical use of theory could meet the ‘cameo character’ criteria. Brown et al.’s recommendation to be deliberate and explicit about the role we give theory and the reason for our choice of theory, and to use this information in deciding how and where theory should appear in a research paper, is useful guidance for how to give theory greater prominence in our work [15]. Secondly, it is important to acknowledge the risks of misapplication of theories to health professions education and encourage a solid understanding of the knowledge and methods of using theories in QIPS education [60, 61].

The limitations of this review are that we may have missed articles due to the range of terms that characterize the QIPS field and our search strategy. We aimed to be consistent about what we counted as ‘theory’, but it is possible that we excluded an article because we identified it as being a learning framework or a superficial use of theory. Given the diverse types of theories as well as differences across disciplines in how theory and associated concepts are defined [9], there is likely some blurriness in the boundaries of what was included and excluded. We made difficult decisions about articles such as those using the concepts of formal, informal and hidden curricula or examinations of QI learning environments [62, 63] that are valuable contributions to the field, but we did not interpret as meeting inclusion criteria. However, we hope that this scoping review is helpful in offering an initial outline of the landscape of the use of theory in QIPS education and future directions. QIPS educators and researchers can seek out papers about theory that could help guide their education planning and research. Alternatively, teams of individuals with expertise in education, QIPS, research and theory would enable collaborative approaches for ensuring rigorous and relevant QIPS education and research. Ultimately, the goal should be for QIPS education and research to continue to inform each other through a meaningful and thoughtful grounding in theory.

## Supplementary Information

Appendix, Part One: Database search strategy

Appendix, Part Two: List of systematic reviews used in search strategy

Appendix, Part Three: Preferred Reporting Items for Systematic reviews and Meta-Analyses extension for Scoping Reviews (PRISMA-ScR) Checklist

Fig. S1: Flow diagram of included studies in scoping review of use of theory in quality improvement and patient safety education

Table S1: Articles included in scoping review of use of theory in quality improvement and patient safety (QIPS) education

Table S2: Types of theories that were mentioned in articles identified as superficial mention of theory and not eligible for inclusion in scoping review of use of theory in quality improvement and patient safety education

Table S3: Definitions and applications of learning and social science theories used in articles included in scoping review of use of theory in quality improvement and patient safety (QIPS) education
