# Supplementary material for: Theory in quality improvement and patient safety education: A scoping review
Source: Perspect Med Educ. 2021 Oct 5;10(6):319–26. doi: 10.1007/s40037-021-00686-5 (PMC8633332; doi:10.1007/s40037-021-00686-5)
Supplement: Supplementary file 1 — Appendix, Part One: Database search strategy [file 40037_2021_686_MOESM1_ESM.docx]

**Appendix, Part One: Database search strategy**

Pubmed:

(curriculum[mesh] or curricul*[tiab]) AND ((patient safety[mesh] or patient safety[tiab]) OR (quality improvement[mesh] or quality improvement[tiab])) AND review[tiab]

EMBASE:

| Searches | Results | Type |
| --- | --- | --- |
| 1 | quality improvement.mp. or exp total quality management/ | 88420 |
| 2 | patient safety.mp. or exp patient safety/ | 140970 |
| 3 | exp curriculum development/ or exp curriculum/ or curriculum.mp. | 112947 |
| 4 | curricula.mp. | 18914 |
| 5 | 1 or 2 | 221919 |
| 6 | 3 or 4 | 119693 |
| 7 | review.m_titl. | 575910 |
| 8 | 5 and 6 and 7 | 126 |

ERIC:

"quality improvement" AND medical
