# Supplementary material for: Theory in quality improvement and patient safety education: A scoping review
Source: Perspect Med Educ. 2021 Oct 5;10(6):319–26. doi: 10.1007/s40037-021-00686-5 (PMC8633332; doi:10.1007/s40037-021-00686-5)
Supplement: Supplementary file 2 — Appendix, Part Two: List of systematic reviews used in search strategy [file 40037_2021_686_MOESM2_ESM.docx]

**Appendix, Part Two: List of systematic reviews used in search strategy**

1. Boonyasai RT, Windish DM, Chakraborti C, Feldman LS, Rubin HR, Bass EB. Effectiveness of teaching quality improvement to clinicians: A systematic review. JAMA. 2007;298:1023-37.
2. Jones AC, Shipman SA, Ogrinc G. Key characteristics of successful quality improvement curricula in physician education: A realist review. BMJ Qual Saf. 2015;24:77-88.
3. Patow CA, Karpovich K, Riesenberg LA, et al. Residents' engagement in quality improvement: A systematic review of the literature. Acad Med. 2009;84:1757-64.
4. Armstrong L, Shepherd A, Harris F. An evaluation of approaches used to teach quality improvement to pre-registration healthcare professionals: An integrative review. Int J Nurs Stud. 2017;73:70-84.
5. Wong BM, Etchells EE, Kuper A, Levinson W, Shojania KG. Teaching quality improvement and patient safety to trainees: a systematic review. Acad Med. 2010;85:1425-39.
6. Abbas MR, Quince TA, Wood DF, Benson JA. Attitudes of medical students to medical leadership and management: A systematic review to inform curriculum development. BMC Med Educ. 2011;11:93.
7. Gordon M, Darbyshire D, Baker P. Non-technical skills training to enhance patient safety: a systematic review. Med Educ. 2012;46:1042-54.
8. Kiersma ME, Plake KS, Darbishire PL. Patient safety instruction in US health professions education. Am J Pharm Educ. 2011;75:162.
9. Kirkman MA, Sevdalis N, Arora S, Baker P, Vincent C, Ahmed M. The outcomes of recent patient safety education interventions for trainee physicians and medical students: A systematic review. BMJ Open. 2015;5:e007705.
10. Medbery RL, Sellers MM, Ko CY, Kelz RR. The unmet need for a national surgical quality improvement curriculum: A systematic review. J Surg Educ. 2014;71:613-31.
11. Nie Y, Li L, Duan Y, et al. Patient safety education for undergraduate medical students: a systematic review. BMC Med Educ. 2011;11:33.
12. Ogrinc G, Headrick LA, Mutha S, Coleman MT, O’Donnell J, Miles PV. A framework for teaching medical students and residents about practice-based learning and improvement, synthesized from a literature review. Acad Med. 2003;78:748-56.
13. Starr SR, Kautz JM, Sorita A, et al. Quality improvement education for health professionals: A systematic review. Am J Med Qual. 2016;31:209-16.
14. Tella S, Liukka M, Jamookeeah D, Smith NJ, Partanen P, Turunen H. What do nursing students learn about patient safety? An integrative literature review. J Nurs Educ. 2014;53:7-13.
15. Windish DM, Reed DA, Boonyasai RT, Chakraborti C, Bass EB. Methodological rigor of quality improvement curricula for physician trainees: A systematic review and recommendations for change. Acad Med. 2009;84:1677-92.
16. Wong BM, Levinson W, Shojania KG. Quality improvement in medical education: Current state and future directions. Med Educ. 2012;46:107-19.
17. Zaugg B, Wangler M. A model framework for patient safety training in chiropractic: A literature synthesis. J Manipulative Physiol Ther. 2009;32:493-9.
18. Bedgood AL, Mellott S. The role of education in developing a culture of safety through the perceptions of undergraduate nursing students: An integrative literature review. J Patient Saf. 2018 Oct 31.
19. da Silva AMB, Bim LL, Bim FL, et al. Patient safety and infection control: Bases for curricular integration. Rev Bras Enferm [Internet]. 2018;71(3):1170-7.
20. Peiris-John R, Selak V, Robb G, et al. The state of quality improvement teaching in medical schools: A systematic review. J Surg Educ; 2020;77(4):889-904.
