# Supplementary material for: Theory in quality improvement and patient safety education: A scoping review
Source: Perspect Med Educ. 2021 Oct 5;10(6):319–26. doi: 10.1007/s40037-021-00686-5 (PMC8633332; doi:10.1007/s40037-021-00686-5)
Supplement: Supplementary file 4 — Fig. S1: Flow diagram of included studies in scoping review of use of theory in quality improvement and patient safety education [file 40037_2021_686_MOESM4_ESM.docx]

**Fig. S1: Flow diagram of included studies in scoping review of use of theory in quality improvement and patient safety education**

Articles of studies included in the 20 systematic reviews of QI/PS education

(n = 560)

Articles that cited one of
the 20 systematic reviews
of QI/PS education

(n = 971)

Articles after removing duplicates

(n = 1097)

Titles and abstracts screened

(n = 1126)

14 Excluded

3 - Irretrievable titles/abstract excluded

11 - Systematic reviews that we used to

identify potentially eligible articles

579 Excluded

458 - Not a curriculum

43 - PS was just rationale for curriculum

35 - Curriculum not at all related to QI/PS

41 - Curriculum focused on building skills

relevant to QI but not focused on QI

concepts

2 - QI was used to improve education process

Full text reviewed

(n = 547)

Additional articles of studies included in 5 reviews identified through initial search

(n = 43)

523 Excluded

249 - No theory

149 - Not a curriculum

102 - Theory mentioned too superficially

6 - Curriculum not at all related to QI/PS

3 - PS was just rationale for curriculum

3 - Not in English

3 - QI was used to improve education process

3 - Curriculum focused on building skills

relevant to QI but not focused on QI

concepts

2 - No full text available

3 - Duplicate (same study)

1 - Not health-professions education of

interest

Articles included

(n = 28)

Articles found hand searching reference list of included studies and relevant journals

(n = 4)
