# Supplementary material for: Theory in quality improvement and patient safety education: A scoping review
Source: Perspect Med Educ. 2021 Oct 5;10(6):319–26. doi: 10.1007/s40037-021-00686-5 (PMC8633332; doi:10.1007/s40037-021-00686-5)
Supplement: Supplementary file 5 — Table S1: Articles included in scoping review of use of theory in quality improvement and patient safety (QIPS) education [file 40037_2021_686_MOESM5_ESM.docx]

**Table S1: Articles included in scoping review of use of theory in quality improvement and patient safety (QIPS) education**

| Authors (Year of Publication), Journal | Country | Profession and setting | QI or PS | Curriculum details | Theory used | Theory used for design or study | Study design and objective | Reported key findings |
| --- | --- | --- | --- | --- | --- | --- | --- | --- |
| Ambrose & Ker (2014), Advances in Health Sciences Education | United Kingdom | Medical students from 5-year university MBChB course | PS | Patient safety learning over 5-year curriculum.  Year 1: ward-based patient safety activities aligned to year 1 clinical skills program  Years 2/3: used patient safety tools as part of ward based clerking activities  Year 5: ward simulation exercise, managed a 3-bedded bay for 20 minutes | Realist evaluation and Mezirow's theory of transformative learning | Study | Realist evaluation to identify and explore mechanisms involved in patient safety learning over a 5-year curriculum and testing for associations between measures of the mechanisms and learning outcomes | A realist approach identified two different levels of reflection that were associated with different patient safety outcomes. Critical reflection was associated with attitudes and reflection was associated with knowledge of actions and error behaviours. |
| Bergh, Bac, Hugo, *et al.* (2016), BMC Medical Education | South Africa | Medical students located across nine clinical learning centres | QI | QI projects provided opportunity for transformation of healthcare and for transformative learning. Oral and written orientation to QI projects at beginning of each rotation and onsite mentors throughout rotation. Submitted group report at end of rotation. | Mezirow’s theory of transformative learning | Study | Qualitative research to explore undergraduate medical students' experiences of their involvement in quality improvement projects during a district health rotation | Quality improvement projects provided an opportunity for both the transformation of health care and for transformative learning, with individual and ‘collective’ self-authorship. |
| Brown, Nidumolu, Stanhope, Koh, Greenway, Grierson (2018), BMJ Quality & Safety | Canada | Medical students from one university | QI | In the Program for Improvement in Medical Education (PRIME) students participated in a facilitated workshop that introduced fundamentals of QI, and then in small groups identified a quality gap in their own medical education and use Model for Improvement to propose a small-scale intervention to address gap. The objectives were to facilitate early acquisition of basic QI knowledge that is applicable in the clinical context and to highlight the importance of QI in medical practice so that participants were encouraged to pursue future QI training. | Cognitive transfer | Design | Sequential explanatory mixed-methods approach to examine the impact of PRIME on first-year medical students and the use of QI in the context of education. | Participation in PRIME increased knowledge of, and comfort with, fundamental QI concepts, even when applied to clinical scenarios. Participants felt that education provided a meaningful context to learn QI and were motivated to participate in future QI projects to drive real-world improvements in the health system. |
| Cresswell, Howe, Steven, *et al.* (2013), BMJ Quality & Safety | United Kingdom | Medical, nursing, pharmacy and physiotherapy students from eight pre-registration university courses | PS | Study investigated professional patient safety knowledge acquired in academic, organizational and practice contexts of healthcare professional preregistration university courses. | Eraut’s theory of formal and informal acquisition of professional knowledge | Study | Qualitative case study to explore how students learn about patient safety | Patient safety was either implicit in curricula or explicitly identified in a limited number of discrete topic areas, with topics reflecting the professional group involved. Patient safety was more visible on placements, but students had limited exposure to positive role models. |
| DeBourgh (2012), Journal of Professional Nursing | United States | Nursing students from a university and a tertiary medical center | PS and QI | Curriculum aimed to increase awareness and knowledge among nursing students and agency staff of the professional accountabilities for patient safety and quality outcomes and to promote application of safety and quality knowledge to nurse-managed patient care. Semester-long clinical rotation involved nursing agency leader briefing with students on safety and quality initiatives, professional accountabilities, nursing care-sensitive patient outcomes and agency performance metrics and monitoring methods, and encouraging dialogue about patient safety topics (e.g., communication, nurses as advocates for PS). | Schon’s reflective learning theory | Design | Survey study to measure students’ knowledge of patient safety and quality standards and to solicit students' perceptions of team behaviours and communication effectiveness before and after participation in the Synergy Partnership | Survey data demonstrated moderate to large effect sizes in gains for safety and quality knowledge and for students’ increased confidence in their impact on patient care outcomes. |
| de Feijter, de Grave, Dornan, *et al.* (2011), Advances in Health Sciences Education | Netherlands | Medical students from one university | PS | Senior clerkship, the final year of a 6-year undergraduate medical curriculum consisted of two 18 week electives: (1) participation in research; (2) a senior clerkship in a department of the student’s choice with students’ responsibilities at an intermediate level between junior clerkships and residency. | Activity theory | Study | Qualitative study to examine how final year medical students learn about patient safety during clinical practice workplace based learning | Results demonstrated that students encounter contradictions when learning to be a doctor and delivering safe patient care. |
| de Feijter, de Grave, Hopmans, *et al.* (2012), Medical Teacher | Netherlands | Medical students from one university | PS | Course designed to learn from personal experiences using reflective learning to change students’ awareness of and attitudes towards patient safety, in order to change students’ behaviour. Course consisted of: 1.5 hours interactive lecture on patient safety (e.g., human factors, systems thinking, incident reporting); preparation of a presentation on patient safety topic (e.g., diagnostic error, communications, disclosure) as well as writing up and reflection on three patient safety incidents; and then 10-minute presentations. | Reflective learning and theory of planned behaviour | Design and Study | Mixed method evaluation to examine how students perceive the conditions for reflective learning, and whether transfer of learning occurs based on reported changes in students' awareness, behavioral intentions and behavior | Students reacted positively towards many elements of the course; their increased knowledge about patient safety topics resulted in a change of attitudes towards these subjects and an increase in awareness of patient safety. This influenced students’ behavioural intention and their behaviour. |
| Gaupp, Dinius, Drazic, et al. (2019), PLOS One | Germany | Medical students from one university | PS | E-learning course used a case-based interactive approach that focused on teamwork, error management, situational awareness, and crisis resource management. The course consisted of three modules and groups of 6 students fulfilled a number of assignments including multiple choice tests, video analysis and case studies. | Theory of attitude-relevant knowledge | Study | Controlled, quasi-experimental study with repeated measures to determine the educational benefits of e-learning on patient safety | Students in the intervention group had significant improvements in technical knowledge and felt better prepared for safe patient practice, with sustained improvements after one year. There was no sustainable significant effect on attitudes towards patient safety. |
| Gaupp Fabry, Korner, (2018), International Journal of Medical Education | Germany | Medical students from one university | PS | E-learning course consisted of two modules, one each on teamwork and error management. Course included journal articles, videos, interactive quizzes, and podcasts. Students worked in small groups and used etherpads and discussion boards for discussions with peers and tutors. Students had online quizzes and had to complete five assignments; two were multiple choice tests and three were designed to foster reflection. | Self-regulated learning and Mezirow’s theory of transformative learning and critical reflection | Study | Cross-sectional survey study using an online survey to explore the influence of critical thinking, self-regulated learning and system usability on the acceptance of e-learning on patient safety | Reflection and learning skills are important factors for e-learning acceptance, but perceived relevance and system usability play a more important role. |
| Goldman, Kuper, Whitehead, et al. (2020), Advances in Health Sciences Education | Canada | Healthcare professionals and administrators working in an academic or community-based healthcare organization participating in one of the three education programs based in a university or hospital setting. | QI & PS | Three advanced post-licensure QI and PS training programs: a Masters, a Certificate and a Fellowship program. All education programs covered a similar range of topics (e.g. QI methods, patient safety, leading and managing change, health policy) but differed in depth given varied number of sessions/courses and program durations. Each education program involved a QI project and coaching. | Sociology of professions: professional socialization, hierarchy and boundaries | Study | Qualitative case study to examine the interprofessional and multiprofessional processes and outcomes of three QI education programs. | Professional socialization, hierarchies and boundaries influence healthcare professionals’ QI attitudes, understandings and practices and therefore need to be understood in the selection of a multiprofessional and/or interprofessional approach in QI education and in the content taught, to support the impact of education on practice as well as the targeting of structural factors beyond the education setting. |
| James, Beattie, Shepherd, *et al.* (2016), Nurse Education in Practice | United Kingdom | Nursing students from one university located at approximately 200 practice placement areas | QI | QI theoretical content was integrated throughout 3-year curriculum to prepare students to devise and test various aspects of a small scale QI project during a seven week clinical placement; students then submitted a report. | Senninger's theory of learning | Study | Qualitative research to explore student nurses' experiences of conducting a QI project while on clinical placement | Students described three themes: time, fear and transformation. Highly structured nature of the QI practicum and support mechanisms needed to buffer the effects of students in the panic zone and to enable many to stay in the discomfort zone, enabling change and learning. |
| Jansma, Wagner & Bijnen (2010), BMC Health Services Research | Netherlands | Medical residents and medical graduates not in training to become specialists from five hospitals | PS | Curriculum aimed at increasing knowledge, attitudes and skills to recognize and cope with unintended events and unsafe situations in an early stage. Format included plenary sessions and small group sessions for a total of 16 hours. Topics included principles of patient safety, human factors, teamwork, medico-legal aspects of patient safety. | Theory of planned behaviour | Study | Qualitative research to investigate residents' intentions and actions to improve patient safety after they received patient safety education | Residents reported various intentions to contribute to patient safety improvement. Numerous actions were taken but there was still a discrepancy between intentions and actual behaviour. |
| Jansma, Zwart, Leistikow, *et al.* (2010), BMC Health Services Research | Netherlands | Specialty registrars from a geographical district | PS | Curriculum aimed to increase specialty registrars’ knowledge, attitudes and skills to recognize and cope with unintended events and unsafe situations at an early stage. The two-day curriculum consisted of plenary session, group discussions, case presentations, experiential learning in small groups, role play, reflection, summative knowledge test. Topics included human error, proceeding after an incident, medico-legal aspects of critical incidents; learning from errors etc. | Theory of planned behaviour | Study | Pre-post questionnaire-based study to investigate the impact of course on specialty registrars’ attitudes, intentions and behaviour towards the voluntary reporting of incidents | There were significant changes in incident reporting attitudes and intentions immediately after the course and during follow-up. However, no significant changes were found in incident reporting behaviour. |
| Jha, Buckley, Gabe, *et al.* (2015), BMJ Quality & Safety | United Kingdom | Junior doctors from five centres part of a foundation school. | PS | Curriculum used emotional stories from patients to enhance the learning experience and provide learners with a greater understanding of safety from the patient’s perspective. The two one-hour sessions consisted of a patient narrative followed by facilitated discussion between patients and junior doctors. The control group received standard faculty-delivered teaching on patient safety with small group discussion. | Mezirow’s theory of transformative learning | Design | Randomized controlled trial to measure impact of patient narratives used to train junior doctors in patient safety | Intervention impacted on emotional engagement and learning about communication but it was uncertain whether these impacts would translate into improved behaviours in the clinical context or if there are negative effects of the intervention. |
| Kaminski, Britto, Schoettker, *et al.* (2012), BMJ Quality & Safety | United States | Faculty members, management and staff members from Division of Patient Services Administration, and administrative and support management and staff from one hospital medical center | QI | Curriculum aimed to develop participants’ knowledge and skill to conduct and lead improvement, get results on a project, and develop a common language and culture/behaviours. The 12 in-class training days over 6 months involved lectures, case studies, interactive application exercises and dialogue, participant reports and assigned readings, project presentations and feedback and one on one coaching. | Kolb's experiential learning theory | Design | Use of surveys, verbal debriefings, QI knowledge self assessments, peer feedback from project reviews, QI director project ratings and project results to assess course using Kirkpatrick’s four-level model for evaluating training programs | Positive improvements in participants’ evaluations of satisfaction, learning, application, impact and value. Improvements in knowledge of QI and project measurable improvements with spread to other units. Graduates continued to lead or participate in QI initiatives. |
| Kiersma, Darbishire, Plake, *et al.* (2009), American Journal of Pharmaceutical Education | United States | Pharmacy students from one university | PS | Laboratory designed to integrate didactic coursework and observations seen in actual practice settings into a skills-based laboratory focused on the prevention of medication errors. The goal was to provide active learning experiences in the recognition, resolution, and prevention of medical errors. Curriculum included medication error-prone prescription processing and counselling simulations, role-playing communication strategies after a medical error occurred, and discussions of an introductory pharmacy practice experience focused on prescription processing and prevention of medication errors. | Self efficacy theory | Study | Pre-post study to evaluate the impact of a first-year skills-based laboratory on students' knowledge and confidence in detecting, preventing, resolving, and communicating medication errors | Students’ awareness of the pharmacist’s role in medication error reduction improved and confidence in their ability to recognize, prevent, and communicate medication errors increased |
| Langer, Martinez, Browning, *et al.* (2016), BMJ Quality & Safety | United States | Physicians, nurses, nurse practitioners, nurse managers, social workers administrators, students, medical interpreters and patients and families from two academic medical centres | PS | Curriculum aimed to develop patient-centred medical error disclosure communication skills for clinicians, and to partner more effectively with patients to prevent errors by supporting patients and families to speak up about concerns. The workshop included didactic teaching, simulation of a medical error disclosure following by debriefing, trigger videos, case-based learning, group discussion. | Psychological safety | Design | Pre-post survey study to assess the feasibility and acceptability of a patient-teacher medical error disclosure and prevention training model | Bringing patients and families and clinicians together to learn about medical error disclosure and prevention was feasible and acceptable to participants. |
| Mak, Miflin. (2012), Medical Teacher | Australia | Medical students from one university | QI | Curriculum aimed to teach students complexities of conducting an audit in professional practice. During the first two years of problem-based learning curriculum, students learned the concepts of evidence-based medicine and QI. In Year 4 students undertook the clinical audit programme that required students to complete an audit using 20-30 patient cases. They were provided with a clinical audit handbook, advised to re-visit prior learning on clinical audit, and participated in lectures, web-based resources, question and answer sessions, panel discussion with health service personnel, peer assessment, and web-based discussion. Students disseminated results and recommendations to stakeholders, and kept a reflective journal | Reflection on learning & community of practice | Design | Post-experience questionnaires and data from curricular documents to assess the educational worth of the clinical audit programme. | The clinical audit programme demonstrated multi-dimensional benefits for students in terms of learning the complexities of conducting an effective audit in professional practice, and for health services in terms of facilitating QI. |
| Nordin, Areskoug-Josefsson. (2020), Cogent Business & Management | Sweden | Multidisciplinary practitioners and decisions-makers in the welfare sector (including physiotherapy, medical and nursing professionals) who are students of a Masters program at one university | QI | Curriculum of the masters program on quality improvement and leadership was based on the Institute for Healthcare Improvements eight knowledge domains for the improvement of healthcare and aimed to improve the welfare sector which includes services for children and the elderly, social care, education and healthcare. The blended learning approach consisted of education occurring online and on campus. Students initiated, designed, led and studied QI projects in their workplaces. | Mezirow’s transformative learning theory | Study | Qualitative interview study to explore alumni’s experienced effects of a master’s program on quality improvement and leadership in the Swedish welfare sector on micro, meso and macro levels. | The master’s program influenced change at micro, meso and macro levels. Theory used to explain personal effects of the program (i.e. greater courage, self-esteem, and capability to see things from different perspectives) that were not included in the original purpose of the study. |
| Ogrinc, Ercolano, Cohen, *et al.* (2014), Academic Medicine | United States | Medical students, residents and attending physicians at a medical centre | QI | Curriculum aimed to integrate teaching, learning and doing QI into the routine clinical work of inpatient internal medicine teams. Didactic teaching and experiential QI project work integrated into 4-week clinical work rotation on the inpatient internal medicine service. | Realist evaluation | Study | Realist evaluation to understand the factors that contribute to residents' engagement in QI work, and determine the educational and care delivery system design factors that facilitate and inhibit the integration of a QI curriculum into the routine work of inpatient resident teams | Three domains affected the delivery of the QI curriculum and engagement of residents in QI work: setting, learner and teacher. Mechanisms included routine updates of team QI work in easy to see locations, protected time for faculty teachers and for research assistant, and QI handoffs that occur resident to resident at end of rotation. |
| Saturno (1995), International Journal for Quality in Health Care | Spain | Physicians and nurses from 30 primary care health centres | QI | Curriculum aimed to provide participants with skills needed to implement a quality evaluation cycle. Participants assigned to either a group that selected problems from their own work environment, a group which had problems for practical exercises proposed by teachers or a group that received no training. Both learning groups participated in one seminar followed by telephone calls every 1-3 months. | Intrinsic motivation | Study | Randomized controlled trial to test the advantages of training, and of a trainee-centered educational strategy, for the implementation of QI activities in health centres | Intervention was more effective in mobilizing the intrinsic motivation and self-determination needs of participants and therefore providing more relevant learning compared to the second group that were taught using teacher-assigned problems and the third group that received no training. |
| Scales, Moin, Fink, *et al.* (2016), International Journal for Quality in Health Care | United States | Medical residents from nine tertiary-care medical center residency training programs at a school of medicine | QI | Online learning platform on quality improvement. Email containing questions on healthcare quality sent over 10 weeks with a total of twenty questions. Participants received feedback. Question repeated in set number of days depending on if answer correct or not, and then retired after two correct responses. Intervention group had specialty-based team assignments with leaderboards to foster competition, and alias assignment to de-identify individual participants. | Spaced education theory and team competition theory | Design | Randomized controlled trial to investigate whether the incorporation of team-based game mechanics into an evidence-based online learning platform could increase resident participation in a QI curriculum | Team competition increased resident participation in the online course. |
| Shaikh, Afsar-Manesh, Amin, *et al.* (2017), International Journal for Quality in Health Care | United States | Medical residents, fellows and physicians from five academic health centres of one university | QI and PS | Online quiz based reinforcement system to teach healthcare quality and patient safety and care transitions. Learners electronically sent multiple-choice questions. Questions covered topics such as: value-based care, healthcare costs, medical errors, transitions of care at hospital discharge, etc. Questions resent at fixed intervals depending on if answered correctly or incorrectly. After submitting answers, learners received immediate feedback, answers, explanations, and references. | Spaced education theory and testing effect | Design | Baseline and final proficiency scores used to test knowledge, and an evaluation survey used to assess the feasibility and acceptability of the online quiz-based reinforcement system | The online quiz-based reinforcement system was feasible, acceptable, and increased knowledge. |
| Shaw, Pernar, Peyre, *et al.*  (2012), BMJ Quality & Safety | United States | Medical interns at two hospitals | PS | Curriculum aimed to improve knowledge and compliance with the Joint Commission National Patient Safety Goals (NPSGs). Online spaced education involved participants receiving 16 case based multiple choice questions and feedback via email in a reinforcing pattern over a number of weeks. This was compared with a more traditional online program that uses an online slide show followed by a multiple choice quiz format. Cases largely based on real life scenarios encountered previously by residents and were selected to illustrate teaching points from the NPSGs covered in the program. Both courses took place over 4-6 weeks. | Spaced education theory | Design | Randomized controlled trial to compare effectiveness of two types of online learning methodologies for improving patient-safety behaviours | While both online methodologies improved knowledge, spaced education was more contextually relevant to trainees and engaging. Spaced education impacted more significantly on both self reported confidence and the behaviour of surgical residents in a simulated scenario. |
| Voogt, van Rensen, van der Schaaf, *et al.* (2016), International Journal for Quality in Health Care | Netherlands | Medical residents from six Dutch teaching hospitals | QI | The Ponder and IMProve (PIMP) program targeted residents’ beliefs and attitudes to QI and leadership in order to demonstrate proactive behaviour. Four 1-hour meetings over one year, part of a larger QI curriculum, where residents discussed opportunities for improvement in their departments and translated them into small-scale QI activities. | Theory of behavioural psychology; self determination theory | Design | Mixed methods study to examine the following outcome measures: organizational awareness, beliefs and attitudes to QI and organizational responsibilities, resident behaviour, barriers and facilitators to successful learning and the program's potential impact on the organization | PIMP sessions made residents more aware of the organizational aspects of their daily work. Residents felt empowered to take up role of change agent. Facilitators included a positive cost-benefit trade-off, valuable group process and safe learning environment. |
| Wittich, Reed, Drefahl, *et al.* (2011), Academic Medicine | United States | Medical residents from an academic medical centre | QI and PS | As part of practice-based learning and improvement curriculum residents completed biannual written reflections on adverse events encountered and their personal practices. Residents prompted to describe events from practice in which care was suboptimal, to reflect on the events from a personal and systems perspective, and then to rate the event’s severity and preventability. Each resident was asked to complete six reflections during three years of training. | Sandars’ critical reflection | Study | Longitudinal study to measure residents’ reflection scores across three years and to determine associations between reflection scores and resident and adverse patient event characteristics | Residents’ reflections on improvement opportunities encountered in practice did not change over three years of training, were lower for systems reflection than for personal reflection and were associated with the preventability of adverse patient events. |
| Wittich, Reed, Drefahl, *et al.* (2011), Medical Education | United States | Medical residents from an academic medical centre | QI | Residents participated in a practice-based learning and improvement curriculum, a component of which required residents to reflect on clinical practice using a structured improvement log, the Mayo Evaluation of Reflection on Improvement Tool (MERIT). Residents also participated in a one-month systems-based practice curriculum. A component of this curriculum was the development of a QI proposal. | Sandars’ critical reflection | Study | Cross-sectional study to explore associations between resident doctors' reflections on QI opportunities and the quality of their QI project proposals | No association between MERIT and Quality Improvement Project Assessment Tool (QIPAT-7) scores indicated a distinction between resident doctors’ skills at reflection on QI opportunities and their abilities to develop QI projects. |
| Wong, Goldman, Goguen, *et al.* (2017), Academic Medicine | Canada | Medical residents and physicians from a Department of Medicine at one university | QI | Co-learning curriculum aimed to develop faculty and residents’ knowledge and skills in QI and to broaden faculty project mentorship and teaching skills specific to QI. Curriculum consisted of prepatory work, two in-person workshops with residents and faculty using didactic and interactive formats, project work over 10 months, and a capstone event where groups present projects. Faculty observe, co-teach, and received feedback on teaching. The program incorporated incentives (e.g. letters for faculty members’ academic teaching dossiers, provision of continuing education credits) for faculty participation. | Bourdieu's social theory of field, habitus and capital | Design | Qualitative research study to examine effectiveness of co-learning, wherein faculty and trainees learn together, as a novel approach for building quality improvement faculty capacity | The incentivizing strategies, in combination with other strategies of the co-learning program, resulted in faculty improvements in knowledge, skill and project facilitation but there was ambivalence about assuming a teacher role. Unplanned outcomes included QI teaching outside of the curriculum, applying QI principles to other work, networking and strengthening one’s QI professional role. |
