# Supplementary material for: Theory in quality improvement and patient safety education: A scoping review
Source: Perspect Med Educ. 2021 Oct 5;10(6):319–26. doi: 10.1007/s40037-021-00686-5 (PMC8633332; doi:10.1007/s40037-021-00686-5)
Supplement: Supplementary file 6 — Table S2: Types of theories that were mentioned in articles identified as superficial mention of theory and not eligible for inclusion in scoping review of use of theory in quality improvement and patient safety education [file 40037_2021_686_MOESM6_ESM.docx]

**Table S2: Types of theories that were mentioned in articles identified as superficial mention of theory and not eligible for inclusion in scoping review of use of theory in quality improvement and patient safety education**

| **Theory** | **Number of articles which mention the type of theory** |
| --- | --- |
| Experiential/Active learning | 57 |
| Adult learning principles | 13 |
| Experiential learning AND Adult learning principles | 15 |
| Experiential learning AND Other* | 6 |
| Other† | 11 |
| Total | 102 |

*Other included realist evaluation, connectionist learning, reflective practice, spaced education

†Other included self determination learning theory, transformative learning theory, complexity theory, theory of planned behaviour, social learning and constructivist theories, constructivist learning theory, cognitive load and self regulation
