# Supplementary material for: Theory in quality improvement and patient safety education: A scoping review
Source: Perspect Med Educ. 2021 Oct 5;10(6):319–26. doi: 10.1007/s40037-021-00686-5 (PMC8633332; doi:10.1007/s40037-021-00686-5)
Supplement: Supplementary file 7 — Table S3: Definitions and applications of learning and social science theories used in articles included in scoping review of use of theory in quality improvement and patient safety (QIPS) education [file 40037_2021_686_MOESM7_ESM.docx]

**Table S3: Definitions and applications of learning and social science theories used in articles included in scoping review of use of theory in quality improvement and patient safety (QIPS) education**

**Learning theories**

| **Theoretical category** | **Theories used in scoping review papers** | **Application to QIPS education** |
| --- | --- | --- |
| **Cognitive learning theories**  Concerned with processes such as perception, insight and meaning. Theories range in their focus on individual learners to the interactions between learners and their environments [1,2]. | **Spaced education theory**  Information presented and repeated over spaced intervals in time increases knowledge, uptake and retention [3].  **Team competition**  A social competition dynamic affects reward-processing neural systems which can have positive and negative effects on engagement and motivation [4].  **Testing effect**  Retention and retrieval of information is increased through learners being tested on content [5]. | Design of spaced online education programs where learners are emailed a scenario and multiple-choice question, receive feedback, and then are sent the same question again at a set timeline depending on response, with question being retired after a set number of correct responses [6-8].  Scales et al. [7] combined spaced online education program with team competition.  Shaikh et al. [8] combined spaced online education program with testing effect. |
|  | **Transfer**  Training that focuses on the fundamental concepts that underpin a skilled process can be effective in promoting transfer of that learning into new contexts, which potentially differ in their outfacing characteristics [9,10]. | Design of a course aimed to increase students’ knowledge of QI in the context of their own education with the aim of them being able to transfer their knowledge to solve problems in the unfamiliar context of the health system [11]. |
|  | **Theory of intrinsic motivation**  An internal force or curiosity that pushes an individual to learn about a particular topic. Learning new knowledge in the context of problems that are meaningful to the learner enhances interest and may foster retrievability and use of knowledge when needed to solve similar problems [12]. | Study of a curriculum using a problem-solving methodology. Involved teaching in relation to quality problems learners identified from their own work environments [13]. |
|  | **Theory of attitude-relevant knowledge**  Under high deliberation conditions, people consider the behavioural relevance and dimensional complexity of knowledge underlying their attitudes before deciding to act on them [14]. | Study of an e-learning course on patient safety to examine whether it can trigger changes in attitude, if the e-learning enables learners to gain specific knowledge and helps them to perceive this knowledge as attitude-relevant [15]. |
|  | **Reflective learning theory (several definitions):**  **“**Reflection for action” is the prospective mental practice of thinking ahead to identify knowledge and/or experience deficits, concerns and anxieties related to performance. “Reflection in action” is thinking on your feet to focus on real-time analysis. “Reflection on action” is retrospective thinking back on actions [16,17].  Reflection as the thinking over of ideas that have been learned, reorganizing them, and considering how they will fit into workplace patterns to improve practice [18]. | Design of communication activities for staff nurses to facilitate nursing students’ reflection about safety and quality during clinical shifts [19].  Design of a course that included medical students’ reflection on patient safety based on their own experiences [20].  Design of a course that included medical students conducting a clinical audit and reflecting on the process of the audit and reactions to results of the audit [21]. |
|  | **Self-regulated learning**  Self-regulated learning requires a learner to regulate three layers: a) the choice of cognitive strategies; b) the use of metacognitive knowledge and skills to direct one’s learning and c) the choice of goals and resources deployed for learning [22]. | Study of the influence of critical thinking, self-regulated learning and system usability on the acceptance of e-learning on patient safety [23]. |
|  | **Kolb’s experiential learning theory**  Kolb’s theory posits that learning occurs when knowledge is created through the transformation of experience. The experiential process involves a four-stage learning cycle (concrete experience, reflective observation, abstract conceptualization, active experimentation) and is affected by the “learning style” (diverging, assimilating, converging, accommodating) through which individual learners engage with material [24]. | Design of a course to develop organizational leaders who can lead and conduct improvement projects. Designed to accommodate all four of Kolb’s learning styles through inclusion of a variety of learning experiences [25]. |
| **Sociocultural learning theories**  Learning is a social process inseparable from the context in which it occurs; learning occurs through dynamic interactions between the individual and the social relations and activities occurring in the learning environment [26]. | **Eraut’s theory of formal and informal learning**  Formal learning is deliberate or organized learning often with dedicated time, presence of a teacher and/or specified outcomes. In contrast, informal learning consists of learning from other people and/or experiences; this type of learning can be implicit, unintended or opportunistic [27,28]. | Study of the formal and informal ways preregistration students in medicine, nursing, pharmacy and physiotherapy learn about patient safety [29]. |
|  | **Senninger’s theory of learning**  Three distinct zones for learning: comfort zone which is largely familiar and where people do not change or learn, a discomfort zone where people feel uncertainty and are most likely to learn, and the panic zone where people are too fearful to learn or change [30]. | Study of nursing students’ experience planning a QI project and their zones for learning in testing aspects of the project in clinical practice [31]. |
|  | **Lave and Wenger’s community of practice**  Novices begin at the periphery of a community by observing and performing basic tasks, and overtime become more central in the community. Through participation, active engagement and assuming increasing responsibility, the individual acquires the roles, skills, norms and values of the community. Learners’ participation in turn transforms the community [26,32]. | Design of a course that involved medical students situated in the clinical environment and engaging learner as an active and collaborative member of the clinical community through performance of a clinical audit [21]. |
| **Transformative learning theories**  Learning that changes the way that learners see the world; it occurs as a result of an experience that encourages reflection, typically challenging preconceived understandings and enabling positive future action [33]. | **Mezirow’s theory of transformative learning**  The process of effecting change in a learner’s frame of reference. Frames of reference refer to associations, concepts, values, feelings and conditioned responses acquired over time that define a person’s worldview and shape expectations, perceptions, cognition and feelings [34]. | Study of medical students’ use of reflective thinking, a process that contributes to transformative learning, in patient safety learning in simulation and ward based patient safety activities showed evidence of both reflection and critical reflection; critical reflection was associated with future intentions [35].  Design of curriculum for junior doctors, that also drew on theories of empathy and moral development, that consisted of reflecting on patient stories and their own experiences, to influence their beliefs, attitudes and intention of future behaviour [36].  Study of students’ QI reports and other documents produced during involvement in QI projects to identify whether project work could lead to transformative learning through action and reflection on actions that challenge current worldviews [37].  Study of the influence of critical thinking, self-regulated learning and system usability on the acceptance of e-learning on patient safety [23].  Study of the influence of a Master’s program on micro, meso and macro levels, which drew upon Mezirow’s transformative theory to explain changes at the personal level that were not the original purpose of the study [38]. |
|  | **Sandars’ critical reflection**  Awareness of one’s own thinking that occurs before, during, and after situations with the aim of developing a greater understanding of one’s self and the situation, to inform future similar situations [39]. | Study of the role of reflection on personal practice to identify how QI methodologies can effect change [40].  Study of associations between resident doctors’ reflection on QI opportunities and quality of their QI project proposals [41]. |
| **Organizational learning theories**  Concerned with how knowledge is created and used within the organization | **Psychological safety**  Psychological safety promotes a safe space for interpersonal risk taking and organizational learning [42]. | Design of an educational program using psychological safety frameworks to develop patient-centered medical error disclosure skills for clinicians and support patients/families to speak up about concerns [43]. |

**Social science theories**

| **Social science field** | **Theories used in scoping review papers** | **Application to QIPS education** |
| --- | --- | --- |
| **Psychology**  The scientific study of mind and behaviour | **Theory of planned behaviour**  Intentions to perform behaviours can be predicted with high accuracy based on an individual’s attitude towards the behaviour, subjective norms, and the perceived behavioural control (ability to control any given behavior). An individual’s intent to perform a behavior is dependent on whether they believe they can be successful [44]. | Study of intentions and actions to improve patient safety following participation in patient safety education courses for residents [45] and specialty registrars [46].  Study to identify the mechanisms that medical students use to learn about patient safety. Questionnaire used to examine personal attitudes, safety in the workplace, personal influence and future intentions [35].  Study used theory of planned behavior to analyze questionnaire and focus group data regarding transfer of patient safety concepts into practice by reported changes in students’ awareness, behavioural intentions and behaviour [20]. |
|  | **Theory of behavioural psychology**  For a behaviour to take place, the underlying beliefs (e.g. self efficacy beliefs, outcome beliefs), facilitators (e.g. training, knowledge and skills) and external barriers (e.g. lack of organizational opportunities) should be addressed to facilitate the intended behaviour [47].  **Self-determination theory**  Study of human motivation and personality that views autonomy, competence and relatedness as three needs that affect motivation [48]. | Design of a program for residents based on theory of behavioural psychology and self- determination theory; program aimed to enhance control beliefs (e.g. feasible QI activities within control), autonomy (e.g. bottom-up contributions) and relatedness (e.g. focus on QI as a group effort) in small-scale QI activities [49]. |
|  | **Self-efficacy theory**  A measure of an individual’s confidence in his/her ability to perform a specific task or behaviour and reach a successful outcome [50]. | Study of the impact of a patient safety laboratory session on recognition, resolution, and prevention of medication errors on students’ self-efficacy, measured through surveys of knowledge regarding medication error prevention and confidence in preventing and resolving errors [51]. |
|  | **Activity theory**  Considers the entire system in which something (such as knowledge acquisition) occurs. This includes the set of interactions, people, tools and rules that exist within complex systems [52]. | Study of how medical students learn about patient safety in the workplace found that they are part of two activity systems with existing contradictions; one focused on learning to be a doctor and the other focused on delivering safe patient care [53]. |
| **Sociology**  The study of human social relationships and institutions. Particularly how human actions and thoughts shape and are shaped by surrounding cultural and social structures. | **Bourdieu’s theoretical concepts of field, habitus and capital**  The concept of “field” describes an arena in which players produce, circulate, and acquire resources that relate to a specific area. These resources, otherwise known as ‘capital’, have different forms. Capital is field specific and confers legitimacy to those who possess more of it. The concept of “habitus” refers to why individuals from a specific group in a given field tend to have predictable patterns of behaviour that are shaped by past experiences and tend to influence future behaviours [54].  **Sociology of professions: professional socialization, hierarchy and boundaries**  Professional socialization: a process characterized by the acquisition of new knowledge and skills along with an altered sense of self, that results in learners coming to think, act and feel like a healthcare professional [55,56].  Professional hierarchy: hierarchy in healthcare has been described as playing out through an occupational and sexual division of labour. Medicine has historically occupied a dominant position in the healthcare division of labour although over time over time there has been tremendous variability and fluidity of boundaries across and within professional groups [57,58].  Professional boundaries: a professional group’s existence occurs in relation to the creation of boundaries with other groups. These boundaries are not static and professionals engage in ongoing activity to maintain, negotiate and expand the boundaries that define their group’s domains of activity [59,60]. | Design of faculty development program that incorporated incentives for faculty participation. This was based on findings from a study that examined how quality improvement is legitimized by different forms of capital in the academic and healthcare delivery fields [61].    Study of interprofessional and multiprofessional processes of three QI education programs using sociology of professions theory with particular attention to professional socialization, hierarchies and boundaries in QI [62]. |
| **Philosophy**  The study of the fundamental nature of knowledge, reality and existence | **Realist evaluation**  Aims to identify what works, for whom and in what circumstances using the formula of Context + Mechanism = Outcome. Mechanisms are often hidden and are sensitive to differences in context. Mechanisms are identified through cyclical investigations involving the examination of patterns and development of plausible theories [63,64]. | Study of mechanisms involved in patient safety learning in a cohort over a 5-year medical school curriculum [35].  Study of factors that contributed to residents’ engagement in QI work, and the educational and care delivery system design factors that facilitated and inhibited the integration of a QI curriculum into the routine work of inpatient resident teams [65]. |

*Articles are cited more than once in instances where more than one type of theory was used.

**References**

1. Mann KV. The role of educational theory in continuing medical education: Has it helped us? J Contin Educ Health Prof. 2004;24 Suppl 1:S22-30.
2. Mann K, MacLeod A. Constructivist: Learning theories and approaches to research. In: Cleland J, Durning SJ, eds. Researching Medical Education. UK: John Wiley & Sons, Ltd.; 2015;51-65.
3. Pashler H, Rohrer D, Cepeda NJ, Carpenter SK. Enhancing learning and retarding forgetting: Choices and consequences. Psychon Bull Rev. 2007;14:187-93.
4. Cikara M, Botvinick MM, Fiske ST. Us versus them: Social identity shapes neural responses to intergroup competition and harm. Psychol Sci. 2011;22:306-13.
5. Carrier M, Pashler H. The influence of retrieval on retention. Mem Cognit. 1992;20:633-42.
6. Shaw TJ, Pernar LI, Peyre SE, et al. Impact of online education on intern behaviour around joint commission national patient safety goals: A randomised trial. BMJ Qual Saf. 2012;21:819-25.
7. Scales CD Jr, Moin T, Fink A, et al. A randomized, controlled trial of team-based competition to increase learner participation in quality-improvement education. Int J Qual Health Care. 2016;28:227-32.
8. Shaikh U, Afsar-Manesh N, Amin AN, Clay B, Ranji SR. Using an online quiz-based reinforcement system to teach healthcare quality and patient safety and care transitions at the University of California. Int J Qual Health Care. 2017;29:735-9.
9. Pass F, Tuovinen J, Tabbers H, Van Gerven P.W.M. Cognitive load measurement as a means to advance cognitive load theory. Educational Psychologist 2003;38:63-71.
10. Kulasegaram K, Min C, Ames K, Howey E, Neville A, Norman G. The effect of conceptual and contextual familiarity on transfer performance. Adv Health Sci Educ Theory Pract 2012;17:489–99.
11. Brown A, Nidumolu A, Stanhope A, Koh J, Greenway M, Grierson L. Can first-year medical students acquire quality improvement knowledge prior to substantial clinical exposure? A mixed-methods evaluation of a pre-clerkship curriculum that uses education as the context for learning. BMJ Qual Saf. 2018;27:576-82.
12. Deci E, Ryan RM. Intrinsic Motivation and Self-Determination in Human Behavior. New York: Plenum Press;1985.
13. Saturno PJ. Training health professionals to implement quality improvement activities. Results of a randomized controlled trial after one year of follow-up. Int J Qual Health Care. 1995;7:119-26.
14. Fabrigar LR, Petty RE, Smith SM, Crites SL Jr. Understanding knowledge effects on attitude-behavior consistency: The role of relevance, complexity, and amount of knowledge. J Pers Soc Psychol. 2006;90:556–77.
15. Gaupp R, Dinius J, Drazic I, Körner M. Long-term effects of an e-learning course on patient safety: A controlled longitudinal study with medical students. PLoS One. 2019; Jan 18;14(1):e0210947.
16. Killion J, Todnem G. A process for personal theory building. Educational Leadership. 1991;48:14-6.
17. Schon D. Educating the Reflective Practitioner. San Francisco: Jossey-Bass; 1987.
18. Moon J. Using reflective learning to improve the impact of short courses and workshops J Contin Educ Health Prof. 2004;24:4-11.
19. Debourgh GA. Synergy for patient safety and quality: Academic and service partnerships to promote effective nurse education and clinical practice. J Prof Nurs. 2012;28:48-61.
20. de Feijter JM, de Grave WS, Hopmans EM, Koopmans RP, Scherpbier AJ. Reflective learning in a patient safety course for final-year medical students. Med Teach. 2012;34:946-54.
21. Mak D.B., Miflin B. Clinical audit in the final year of undergraduate medical education: Towards better care of future generations. Med Teach. 2012;34(4):e251-7.
22. Boekaerts M. Self-regulated learning: where we are today. International Journal of Educational Research. 1999;31:445-57.
23. Gaupp R, Fabry G, Körner M. Self-regulated learning and critical reflection in an e-learning on patient safety for third-year medical students. Int J Med Educ. 2018;9:189-194.
24. Kolb DA. Experiential Learning: Experience as the Source of Learning and Development. Englewood Cliffs, New Jersey: Prentice-Hall Inc.; 1984.
25. Kaminski GM, Britto MT, Schoettker PJ, Farber SL, Muething S, Kotagal UR. Developing capable quality improvement leaders. BMJ Qual Saf. 2012;21:903-11.
26. Mann KV. Theoretical perspectives in medical education: past experience and future possibilities. Med Educ. 2011;45:60-8.
27. Eraut M. Developing Professional Knowledge and Competence. London: Falmer Press; 1994.
28. Eraut M. Informal learning in the workplace. *Studies in Continuing Education.* 2004;26:247-73.
29. Cresswell K, Howe A, Steven A, et al. Patient Safety Education Research Group. Patient safety in healthcare preregistration educational curricula: Multiple case study-based investigations of eight medicine, nursing, pharmacy and physiotherapy university courses. BMJ Qual Saf. 2013;22:843-54.
30. NHS Institute for Innovation and Improvement. Managing the human dimesnions of change. Coventry: NHSIII 2005.
31. James B, Beattie M, Shepherd A, Armstrong L, Wilkinson J. Time, fear and transformation: Student nurses' experiences of doing a practicum (quality improvement project) in practice. Nurse Educ Pract. 2016;19:70-8.
32. Lave J, Wenger E. Situated Learning: Legitimate Peripheral Participation. Cambridge: Cambridge University Press; 1991.
33. Taylor EW. An update of transformative learning theory: A critical review of the empirical research (1999-2005). Int J Lifelong Educ. 2007;26:173-91.
34. Mezirow J. Transformative learning: Theory to practice. New Dir Adult Contin Educ. 1997;74:5-12.
35. Ambrose LJ, Ker JS. Levels of reflective thinking and patient safety: an investigation of the mechanisms that impact on student learning in a single cohort over a 5 year curriculum. Adv Health Sci Educ Theory Pract. 2014;19:297-310.
36. Jha V, Buckley H, Gabe R, et al. Patients as teachers: A randomised controlled trial on the use of personal stories of harm to raise awareness of patient safety for doctors in training. BMJ Qual Saf. 2015;24:21-30.
37. Bergh AM, Bac M, Hugo J, Sandars J. "Making a difference" - Medical students' opportunities for transformational change in health care and learning through quality improvement projects. BMC Med Educ. 2016;16:171.
38. Nordin A, Areskoug-Josefsson K. Effects of a Swedish master’s programme on quality improvement and leadership – A qualitative study on micro, meso and macro levels of the welfare sector. Cogent Business & Management. 2020;7:1725308.
39. Sandars J. The use of reflection in medical education: AMEE Guide No. 44. Med Teach. 2009;31:685-95.
40. Wittich CM, Reed DA, Drefahl MM, et al. Residents' reflections on quality improvement: Temporal stability and associations with preventability of adverse patient events. Acad Med. 2011;86:737
41. Wittich CM, Reed DA, Drefahl MM, et al. Relationship between critical reflection and quality improvement proposal scores in resident doctors. Med Educ. 2011;45:149-54.
42. Edmondson A. Psychological safety and learning behavior in work teams. Admin Sci Q. 1999;44:350-83.
43. Langer T, Martinez W, Browning DM, Varrin P, Sanoff Lee B, Bell SK. Patients and families as teachers: A mixed methods assessment of a collaborative learning model for medical error disclosure and prevention. BMJ Qual Saf. 2016;25:615-25.
44. Ajzen I. The theory of planned behavior. Organ Behav Hum Decis Process. 1991;50:179-211.
45. Jansma JD, Wagner C, Bijnen AB. Residents' intentions and actions after patient safety education. BMC Health Serv Res. 2010;10:350.
46. Jansma JD, Zwart DL, Leistikow IP, Kalkman CJ, Wagner C, Bijnen AB. Do specialty registrars change their attitudes, intentions and behaviour towards reporting incidents following a patient safety course? BMC Health Serv Res. 2010;10:100.
47. Fishbein M, Yzer MC. Using theory to design effective health behavior interventions. Commun Theory. 2003;13:164-83.
48. Ryan RM, Deci EL. Self-determination theory and the facilitation of intrinsic motivation, social development, and well-being. Am Psychol. 2000;55:68-78.
49. Voogt JJ, van Rensen ELJ, van der Schaaf MF, Noordegraaf M, Schneider MM. Building bridges: Engaging medical residents in quality improvement and medical leadership. Int J Qual Health Care. 2016;28:665-74.
50. Kiersma ME, Darbishire PL, Plake KS, Oswald C, Walters BM. Laboratory session to improve first-year pharmacy students' knowledge and confidence concerning the prevention of medication errors. Am J Pharm Educ. 2009;73:99.
51. Bandura A. Self-efficacy mechanism in human agency. Am Psychol. 1982;37:122-47.
52. Engestrom Y. Expansive learning at work: Toward an activity theoretical reconceptualization. J Educ Work. 2001;14:133-56.
53. de Feijter JM, de Grave WS, Dornan T, Koopmans RP, Scherpbier AJ. Students' perceptions of patient safety during the transition from undergraduate to postgraduate training: An activity theory analysis. Adv Health Sci Educ Theory Pract. 2011;16:347-58.
54. Bourdieu P. The forms of capital. In: Richardson JG, ed. Handbook of Theory and Research for the Sociology of Education. New York, NY: Greenwood; 1986:241-58.
55. Hafferty FW. Professionalism and the socialization of medical students. In Cruess RL, Cruess SR, & Steinert Y, eds. Teaching Medical Professionalism: Supporting the Development of a Professional Identity. Cambridge: Cambridge University Press;2009;53-70 .
56. Merton, R. K. (1957). Some preliminaries to a sociology of medical education. In R. K. Merton, G. G. Reader, & P. L. Kendall (Eds.), *The student physician: Introductory studies in the sociology of medical education*. Cambridge: Harvard University Press.
57. Willis, E. (1989). *Medical dominance: The division of labour in Australian health care*. Sydney: Allen & Unwin.
58. Nancarrow, S. A., & Borthwick, A. M. (2005). Dynamic professional boundaries in the healthcare work- force. *Sociology of Health and Illness, 27*(7), 897–919.
59. Abbott, A. D. (1988). *The system of professions*. Chicago: University of Chicago Press.
60. Lamont, M., & Molnar, V. (2002). The study of boundaries in the social sciences. *Annual Review of Sociology, 28,* 167–95.
61. Wong BM, Goldman J, Goguen JM, et al. Faculty-resident "Co-learning": A longitudinal exploration of an innovative model for faculty development in quality improvement. Acad Med. 2017;92:1151-9.
62. Goldman J, Kuper A, Whitehead C, et al. Interprofessional and multiprofessional approaches in quality improvement education. Adv Health Sci Educ Theory Pract. 2020; Oct 28.
63. Pawson R. Evidence-based Policy: A Realist Perspective. Thousand Oaks, California: SAGE Publications; 2006.
64. Pawson R, Tilley N. Realistic Evaluation. London: Sage Publications; 1997.
65. Ogrinc G, Ercolano E, Cohen ES, et al. Educational system factors that engage resident physicians in an integrated quality improvement curriculum at a VA hospital: A realist evaluation. Acad Med. 2014;89:1380-5.
